# Conserved Dopamine Neurotrophic Factor-Transduced Mesenchymal Stem Cells Promote Axon Regeneration and Functional Recovery of Injured Sciatic Nerve

**DOI:** 10.1371/journal.pone.0110993

**Published:** 2014-10-24

**Authors:** Yi Liu, Lin Nie, Hua Zhao, Wen Zhang, Yuan-Qiang Zhang, Shuai-Shuai Wang, Lei Cheng

**Affiliations:** 1 Department of Spine Surgery, Qilu Hospital of Shandong University, Jinan, China; 2 Shandong University Qilu Hospital Research Center for Cell Therapy, Key Laboratory of Cardiovascular Remodeling and Function Research, Qilu Hospital of Shandong University, Jinan, China; Universidade Federal do Rio de Janeiro, Brazil

## Abstract

Peripheral nerve injury (PNI) is a common disease that often results in axonal degeneration and the loss of neurons, ultimately leading to limited nerve regeneration and severe functional impairment. Currently, there are no effective treatments for PNI. In the present study, we transduced conserved dopamine neurotrophic factor (CDNF) into mesenchymal stem cells (MSCs) in collagen tubes to investigate their regenerative effects on rat peripheral nerves in an *in vivo* transection model. Scanning electron microscopy of the collagen tubes demonstrated their ability to be resorbed *in vivo*. We observed notable overexpression of the CDNF protein in the distal sciatic nerve after application of CDNF-MSCs. Quantitative analysis of neurofilament 200 (NF200) and S100 immunohistochemistry showed significant enhancement of axonal and Schwann cell regeneration in the group receiving CDNF-MSCs (CDNF-MSCs group) compared with the control groups. Myelination thickness, axon diameter and the axon-to fiber diameter ratio (G-ratio) were significantly higher in the CDNF-MSCs group at 8 and 12 weeks after nerve transection surgery. After surgery, the sciatic functional index, target muscle weight, wet weight ratio of gastrocnemius muscle and horseradish peroxidase (HRP) tracing demonstrated functional recovery. Light and electron microscopy confirmed successful regeneration of the sciatic nerve. The greater numbers of HRP-labeled neuron cell bodies and increased sciatic nerve index values (SFI) in the CDNF-MSCs group suggest that CDNF exerts neuroprotective effects *in vivo*. We also observed higher target muscle weights and a significant improvement in muscle atrophism in the CDNF-MSCs group. Collectively, these findings indicate that CDNF gene therapy delivered by MSCs is capable of promoting nerve regeneration and functional recovery, likely because of the significant neuroprotective and neurotrophic effects of CDNF and the superior environment offered by MSCs and collagen tubes.

## Introduction

Peripheral nervous injury (PNI) is a common form of trauma [1], and the recovery of neural function following PNI depends on the severity of the injury. Following transection of the whole nerve trunk, degenerative events, including the breakdown of axons and myelin, are initiated proximally and distally to the injury site [2]. To restore and reshape the original motor and sensory function, regenerated nerve fibers must pass through the damaged area and reestablish connections with appropriate target organs.

Bringing the proximal and distal ends of the nerve together into closer apposition without tension is necessary for successful nerve repair. If the distance between the proximal and distal stumps is too large to implement end-to-end anastomosis, a nerve graft is required. Autologous nerve grafting surgery appears to be very effective, and its therapeutic outcome is also commendable [3]. Many less important nerves, including the sural nerves, superficial cutaneous nerves, or lateral and medial antebrachial cutaneous nerves, can be harvested as grafts [4]. However, grafting remains problematic, with faults such as the deficit created by graft harvest, the danger of the formation of potentially painful neuromas and the limited availability of autologous tissue. Although axonal regeneration occurs, studies of clinical outcomes show that severe functional deficits persist due to the failure of connection or inappropriate connections [5], [6]. Thus, it is important to identify new approaches for repair after PNI.

Neurotrophic factors (NTFs) that enhance the survival, maintenance, and differentiation of neurons are considered a potential therapeutic approach in a variety of diseases of the central nervous system (CNS) and peripheral nervous system (PNS). A number of experiments have supported the notion that NTFs play important roles in supporting peripheral nerve regeneration, axon remyelination, and protecting both motor and sensory neurons [7]–[9]. Previous studies reported that CDNF and MANF have protective functions toward the midbrain dopaminergic system in models of Parkinson’s disease [10] and toward cortical neurons in models of ischemia [11]. We recently described the anti-inflammatory properties of cerebral dopamine neurotrophic factor (CDNF), which acted in lipopolysaccharide (LPS)-induced microglia by inhibiting c-Jun N-terminal kinase (JNK) signaling [12]. Moreover, CDNF overexpression in astrocytes alleviates cell damage and proinflammatory cytokine secretion [13], which may suggest a novel mechanism of neuroprotection in the CNS and PNS. We found that direct injection of lentiviral (LV)-CDNF could provide durable and stable concentrations of CDNF protein with the potential to enhance peripheral nerve regeneration and promote morphological as well as functional recovery in a rat sciatic nerve transection model [14].

Mesenchymal stem cells (MSCs) may be beneficial in the treatment of neurodegenerative disease [15], nervous system traumatic disease [16], and cerebrovascular diseases [17], although the mechanisms underlying these benefits remain unclear. The ability of MSCs to differentiate into cell lineages such as myocardiocytes [18] and neurons [19] makes them valid candidates for cell-based therapy and tissue engineering. In our previous research, silicone conduits provided a potential alternative for nerve repair without sacrificing sound nerves. However, non-degradable conduits have their own disadvantages because in the late stages of neural regeneration, these conduits may cause nerve compression. Two aspects are considered responsible for the nerve compression: the formation of fibrotic tissue in the inner wall of the conduit and the space limitations of the conduit itself, which is likely to hinder nerve regeneration and require a second surgery for removal. In this study, the extracellular matrix (ECM) molecule collagen, which shows excellent biocompatibility and cell adhesion, may represent a promising mediator for nerve regeneration.

We hypothesized that CDNF supplementation with MSCs therapy immediately post-injury would significantly improve regeneration. We examined the early effects of LV-CDNF injection, which greatly compensates for the relatively low level of CDNF present during regeneration. Our findings indicate that CDNF-transduced MSCs are able to produce relatively high levels of CDNF *in vivo*, remarkably enhance the axon regeneration, promote motor neuron survival, and reduce the atrophy of denervated muscle.

## Materials and Methods

### Ethics Statement

Experimental procedures were approved by the Animal Ethical Committee of Shandong University and followed the guidance of the Animal Management Rules of the Chinese Ministry of Health (document No. 55, 2001). After surgery, the animals were placed in a warm room with food and water ad libitum for recovery and were monitored every 12 hours. All efforts were made to relieve the suffering of the animals and reduce the number of animals used.

### Recombinant CDNF LV vector

Recombinant CDNF LV vector design and production were performed as described previously [13].

### Rat marrow stromal stem cells culture and flow cytometry

**Figure 1:**
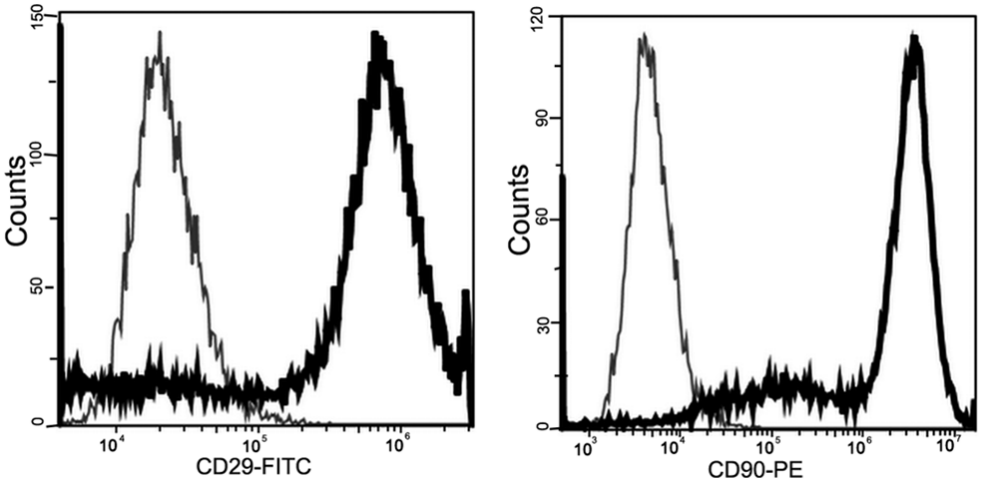
Flow cytometry results. Rat-derived MSCs are positive for CD29 and CD90.

MSCs were prepared from adult male Wistar rats and cultured in Dulbecco’s modified Eagle’s medium (DMEM, Gibco, Grand Island, NY) containing 10% fetal bovine serum (FBS, Gibco) as described previously [20]. More than 60,000 cells were incubated with PE-conjugated anti-rat CD90 and FITC-conjugated anti-rat CD29 (both from Biolegend, San Diego, CA) monoclonal antibodies at room temperature for 15 min while shielded from light. Following a 10-min wash in phosphate-buffered saline (PBS), the labeled cells were detected by flow cytometry using an Accuri C6 Flow Cytometer (BD Biosciences, Franklin Lakes, NJ). FlowJo software (Treestar, Inc., San Carlos, CA) was used to analyze the data. Most MSCs expressed both CD29 and CD90 (Fig. 1).

### Transduction of MSCs with LV vectors

MSCs were cultured in 6-well plates (Corning, Corning, NY) at a density of 5×10^4^/well, and each well contained 1 mL DMEM (5.5 mmol/L glucose) with 10% FBS. Primary MSCs were cultured for 24 h. Viral multiplicity of infection (MOI) was determined with Lenti-X GoStix (Clontech, TaKaRa, Shiga, Japan). MSCs transduced with 5 MOI of the plenti-CDNF/plenti-his LV were used in subsequent experiment. Because plenti-CDNF does not contain a green fluorescent protein (GFP) reporter, the transduction efficiency was detected using western blots.

### Western blot analysis

Protein was extracted and determined using a bicinchoninic acid (BCA) Protein Assay Kit (Sangon Biotech Co., Ltd, Shanghai, China). Samples with equal amounts of total protein (10 µg) were separated using polyacrylamide gel electrophoresis with 5% stacking and 12% separating gels. Proteins were subsequently transferred to polyvinylidene difluoride (PVDF) membranes (Millipore, Billerica, MA) and stained with primary antibodies (goat anti-CDNF, 1∶1000, R & D Systems, Inc., Minneapolis, MN) and a rabbit anti-goat immunoglobulin (IgG)-horseradish peroxidase (HRP) secondary antibody (1∶30,000, Cell Signaling Technology, Danvers, MA). Loading of equal amounts of protein was confirmed by reprobing the membranes with a mouse anti-β-actin -HRP antibody (1∶10,000, Abcam, Cambridge, UK). Protein bands were detected using a FluorChem E Chemiluminescent Western Blot Imaging System (Cell Biosciences, Santa Clara, CA) and quantified by densitometry analysis using ImageJ software (National Institutes of Health, Bethesda, MD).

### Animals

Wistar rats were purchased from the Laboratory Animal Center of Shandong University. Surgical procedures were performed under deep anesthesia induced by an intraperitoneal (i.p.) injection of pentobarbital sodium (20 mg/kg). A surgical microscope was used for surgical intervention under sterile conditions. Animals were sacrificed without suffering using sodium pentobarbital at different time points. The rats were housed under standard conditions in a 12∶12-h light/dark cycle with food and water ad libitum. After surgery, we regularly observed the animals’ health status, including the status of the surgical incision healing, the activities of the operated limbs and the formation of foot ulcers.

### Surgical procedure and application of CDNF-MSCs

Seventy-six male Wistar rats weighing 220–230 g were randomly assigned to the following groups: CDNF-MSCs (animals that received CDNF-transduced MSCs, n = 19), LV-MSCs (animals that received LV-transduced MSCs, n = 19), MSCs (animals that received no transduced MSCs, n = 19), and naive (animals that received DMEM, n = 19). In addition, three rats that did not undergo repair surgeries and fifteen animals that received no repair after nerve transection were used as controls. When the animals were fully unresponsive, the right sciatic nerve was dissected free and transected 1 cm proximal to the trifurcation with a small segment removed to create a 5-mm gap. For animals in the CDNF-MSCs group, 6 µL (containing 1×10^6^ cells) solution was then immediately injected into the transected sciatic nerve stumps with a 10- L syringe (Shanghai Gaoge Industrial and Trading Co., Ltd., Shanghai, China). The LV-MSCs group received the LV transduced MSCs, the MSCs group received the MSCs without the transgenes, and the naive group received DMEM as a negative control. Each animal in the repair groups underwent immediate entubulation repair with an 9-mm-long collagen tube made of highly purified type I collagen extracted from bovine tendon (Beijing TianXinFu Medical Appliance Co., Ltd., Beijing, China) that was inserted 2 mm into the nerve stump and sutured to the tube with 7-0 microsutures, maintaining a 5-mm distance between the proximal and distal stumps.

### HRP tracing method

After 12 weeks, four animals in each group were studied using the HRP tracing method. When the animals were fully unresponsive, the sciatic nerve was exposed again distal to the nerve conduit implant. The distal stump was injected with an HRP solution containing 30% free HRP (Beijing Biosynthesis Biotechnology Co., Ltd, Beijing, China). An additional three animals without previous surgery were processed in the same fashion as a control. After 3 days, the L4–L5 lumbar enlargements of the spinal cord were removed and stored at 4°C for 24 hours in 0.1 M PBS at pH 7.4 containing 30% sucrose. Frozen sections were cut at a thickness of 40 µm, reacted with 3,3,5,5-tetramethylbenzidine (TMB; Bioss, Beijing, China) and mounted on chrome-alum gelatin-coated slides, and then the sections were dehydrated and coverslipped. HRP-containing cells were counted at 100x magnification.

### Histomorphological analysis

The histological assessments were performed at 4, 8 and 12 weeks after surgery. Regenerating cables were cut and carefully separated from the collagen tubes under the microscope. The distal regenerating nerve was embedded, cut longitudinally into 5-m-thick sections and labeled with primary antibodies to neurofilament 200 (NF200; 1∶200, Bioss) and S-100 (1∶200, Bioss). The proximal regenerating nerve was embedded, sectioned into 5-m-thick transverse sections and labeled with primary antibodies to neurofilament 200 (NF200; 1∶200, Bioss). A goat anti-rabbit immunoglobulin (IgG)-HRP secondary antibody (1∶200; Beijing Golden Bridge Biotechnology Co., Ltd, Beijing, China) was applied. The sections were detected with 3, 3-diaminobenzidine (DAB, Abcam Inc., USA), and the sections were then dehydrated and coverslipped. For each slide, six fields were randomly chosen for imaging. Images were captured using a Nikon Eclipse 80i microscope (Nikon, Tokyo, Japan). Image-Pro Plus (IPP) software (Media Cybernetics, Bethesda, MD) was used to quantify the NF200 and S100-positive sites, which were stained brown in pixels at 400x magnification. To ensure that these data were comparable, all images were taken and measured using the same parameters.

At 8 and 12 weeks after surgery, middle regenerating nerve segments were rapidly harvested and fixed in cold buffered 3% glutaraldehyde solution. Ultrathin sections of newly regenerated nerves were observed using transmission electron microscopy (TEM, performed by the Department of Electron Microscopy, Shandong University School of Medicine). A JEM-1200EX (JOEL, Tokyo, Japan) was used to capture the images. At 4, 8 and 12 weeks after surgery, the ultrastructural features of collagen tubes were observed before and after implantation using scanning electron microscopy (SEM, performed by the Department of Electron Microscopy, Shandong University School of Materials Science and Engineering). A SU-70 FE-SEM (HITACHI, Tokyo, Japan) was used to capture the images. We randomly selected six sections for recording and analysis. Six visual fields were selected for each section. The degradation of the tube was estimated based on the thickness of the tube wall at 4, 8, and 12 weeks after implantation. We also evaluated axonal regeneration and degree of myelination using: (1) the diameter of myelinated axons; (2) the thicknesses of myelin sheaths; (3) the ratio of the axon diameter to the fiber diameter (G-ratio).

### Walking track analysis

Walking track analysis was performed as previously described [14], [21]. To measure SFI, we randomly chose five animals in each group for walking track analysis at 2, 4, 6, 8, 10, and 12 weeks after surgery, according to the previously described protocol. Three variables for the feet on the experimental side and the normal side were measured as follows. EPL indicates the operated experimental paw length; NPL, the normal paw length; ETS, the operated experimental toe spread, which represents the distance between the first and fifth toes; NTS, the normal toe spread; EIT, the operated experimental intermediary toe spread, which represents the distance between the second and fourth toes; and NIT, the normal intermediary toe spread. SFI was calculated using the following formula: SFI = –38.3 (EPL–NPL)/NPL+109.5 (ETS–NTS)/NTS+13.3 (EIT–NIT)/NIT–8.8.

### Target muscle weight analysis and Masson’s trichrome staining

After the animals were killed, the gastrocnemius muscles of the operated and normal limbs of five rats in each group were exposed, resected and weighed. For each animals, a gastrocnemius muscle belly section was fixed in 4% paraformaldehyde, cut into 5-µm sections, and stained with Masson’s collagen staining method. The wet weight ratio of the gastrocnemius muscle was calculated by comparing the weight of the gastrocnemius muscle from the operated side to that of the normal side. The percentage of muscle fiber was quantitatively analyzed using IPP software.

### Statistical analysis

All histological and functional measures were performed by observers blinded for each group. The data are presented as the means ± standard errors of mean (SEMs). Statistical analysis of data was performed by one-way ANOVA using LSD and Bonferroni’s test for multiple comparisons of means. Differences were considered significant at P<0.05.

## Results

### CDNF expression *in* *vitro* and *in* *vivo*

**Figure 2:**
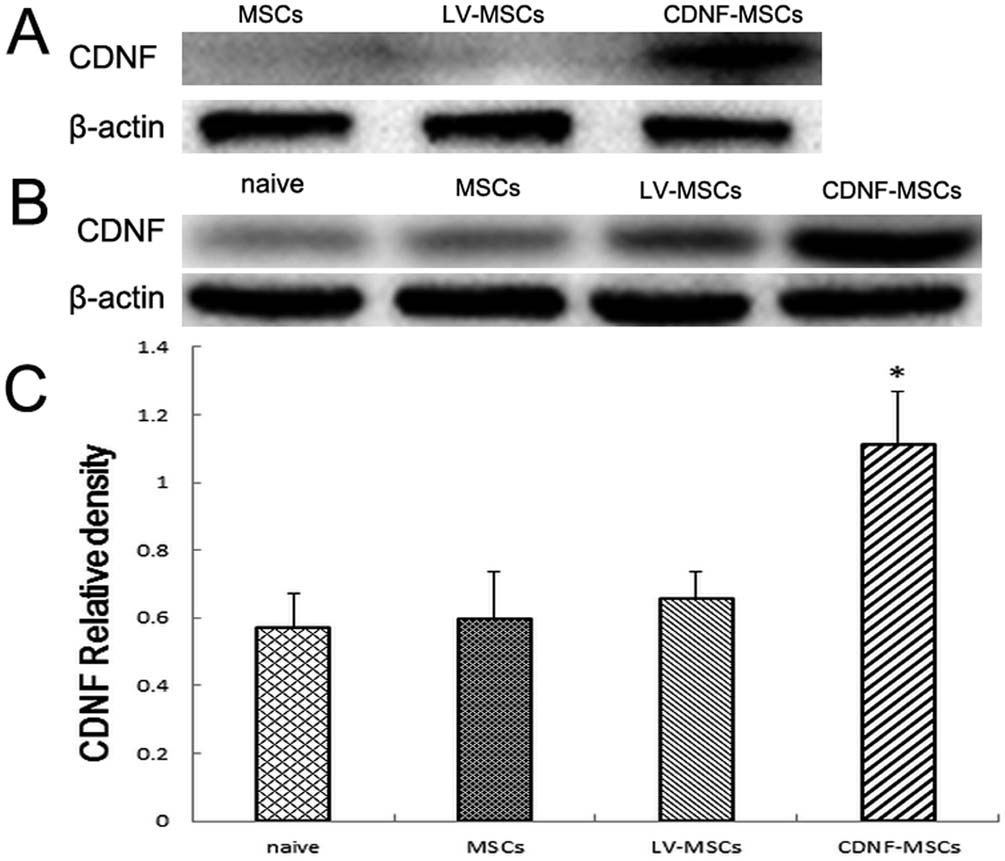
CDNF efficiently transfers and expresses CDNF *in* *vitro* and *in* *vivo*. (A) Western blot of CDNF protein 2 weeks after transduction. (B) Detection of CDNF in the distal sciatic nerve 4 weeks after injury. (C) Identified CDNF production in the distal sciatic nerve as a ratio of the target protein to β-actin.

We performed western blots to assess CDNF production in CDNF-transduced MSCs, LV-transduced MSCs, and nontransduced MSCs 2 weeks after transduction. No CDNF was detected in either LV-transduced MSCs or nontransduced MSCs. However, high levels of CDNF were detected in CDNF-MSCs (Fig. 2A). This increase was due to the CDNF-transduced MSCs and is consistent with our previously studies.

The levels of CDNF following the application of CDNF-MSCs, LV-MSCs, and MSCs were assessed and compared with levels in DMEM-injected rats after sciatic repair. At 4 weeks after injection, the expression of CDNF protein in the CDNF-MSCs group was considerably higher than in the nerves injected with LV- MSCs, MSCs, or DMEM (Fig. 2B). The CDNF levels in the LV- MSCs, MSCs, and DMEM groups were not significantly different (Fig. 2C) (n = 3, p>0.05).

### Collagen tubes are gradually absorbed *in* *vivo*

**Figure 3:**
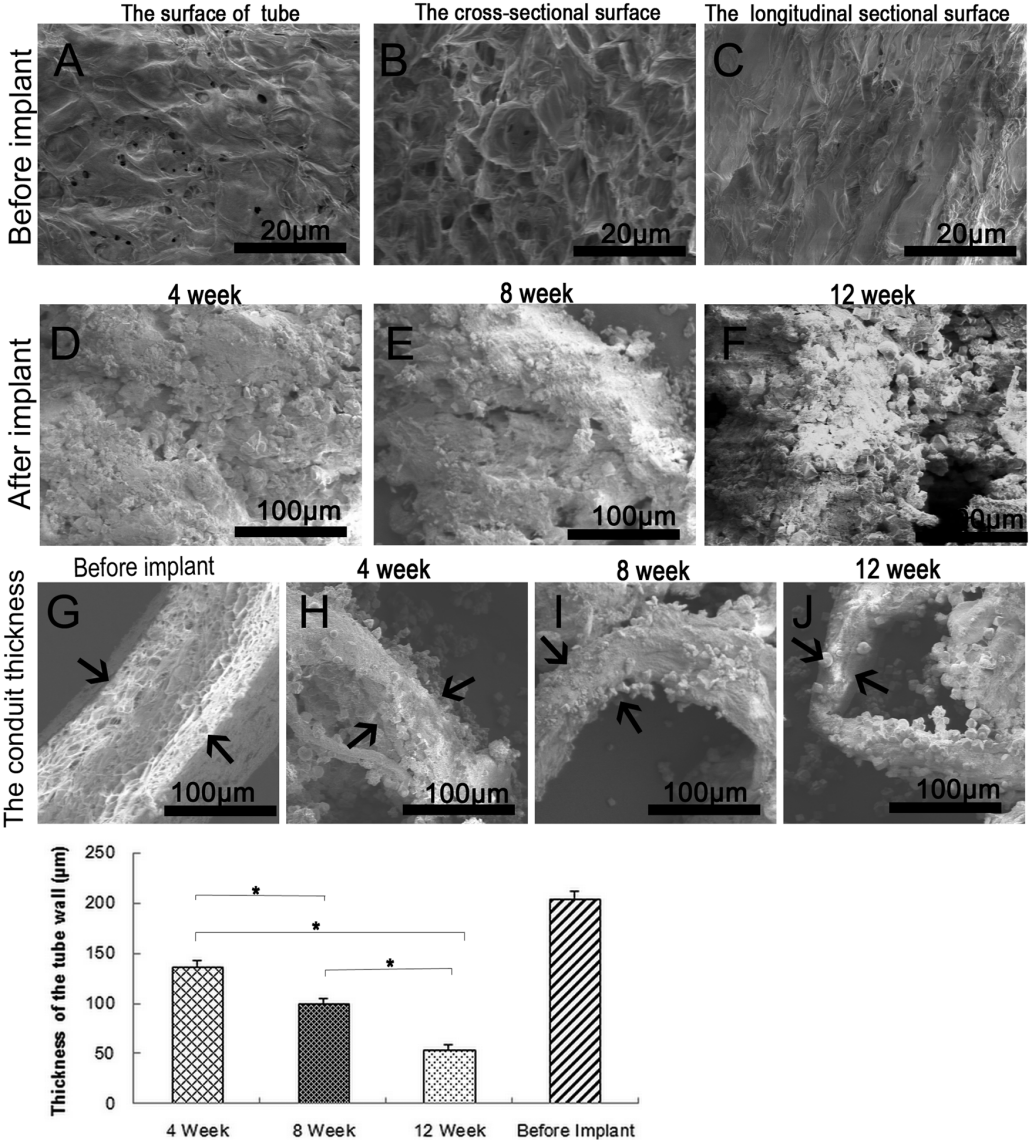
SEM of collagen tubes. (A) The surface of the collagen tube. Scale bar = 20 µm. (B) The cross-sectional surface. Scale bar = 20 µm. (C) The longitudinal section surface. Scale bar = 20 µm. (D–F) The inner surface of tube wall at 4, 8 and 12 weeks after implantation shows that most of collagen tube is absorbed over time. Scale bar = 100 µm. (G) Tube wall thickness before implantation. Scale bar = 100 µm. (H−J) Tube wall thickness at 4, 8, and 12 weeks after implantation, showing that it biodegrades over time. Scale bar = 100 µm. (K) Quantitative analysis of the thickness of the tube wall. n = 5, *p<0.05.

Inspection of the collagen tube before implantation revealed a smooth surface with numerous pores (Fig. 3A). The transverse section surface showed a grid-like structure (Fig. 3B), whereas the longitudinal section surface showed a poriferous and lax structure (Fig. 3C). The tube wall thickness before surgery is shown in Fig. 3G. These characteristics favor nutrient diffusion through the tube wall, creating an advantageous internal microenvironment that enhances nerve regeneration. At 4, 8, and 12 weeks after surgery, we also evaluated modifications of the tube wall with SEM. The surface of the tube residue after absorption revealed that the tube interacted with the nerve growing inside (Fig. 3D–F). The thickness of the tube wall became thinner, demonstrating its biodegradability *in* *vivo* (Fig. 3H–K).

### Histological analysis

**Figure 4:**
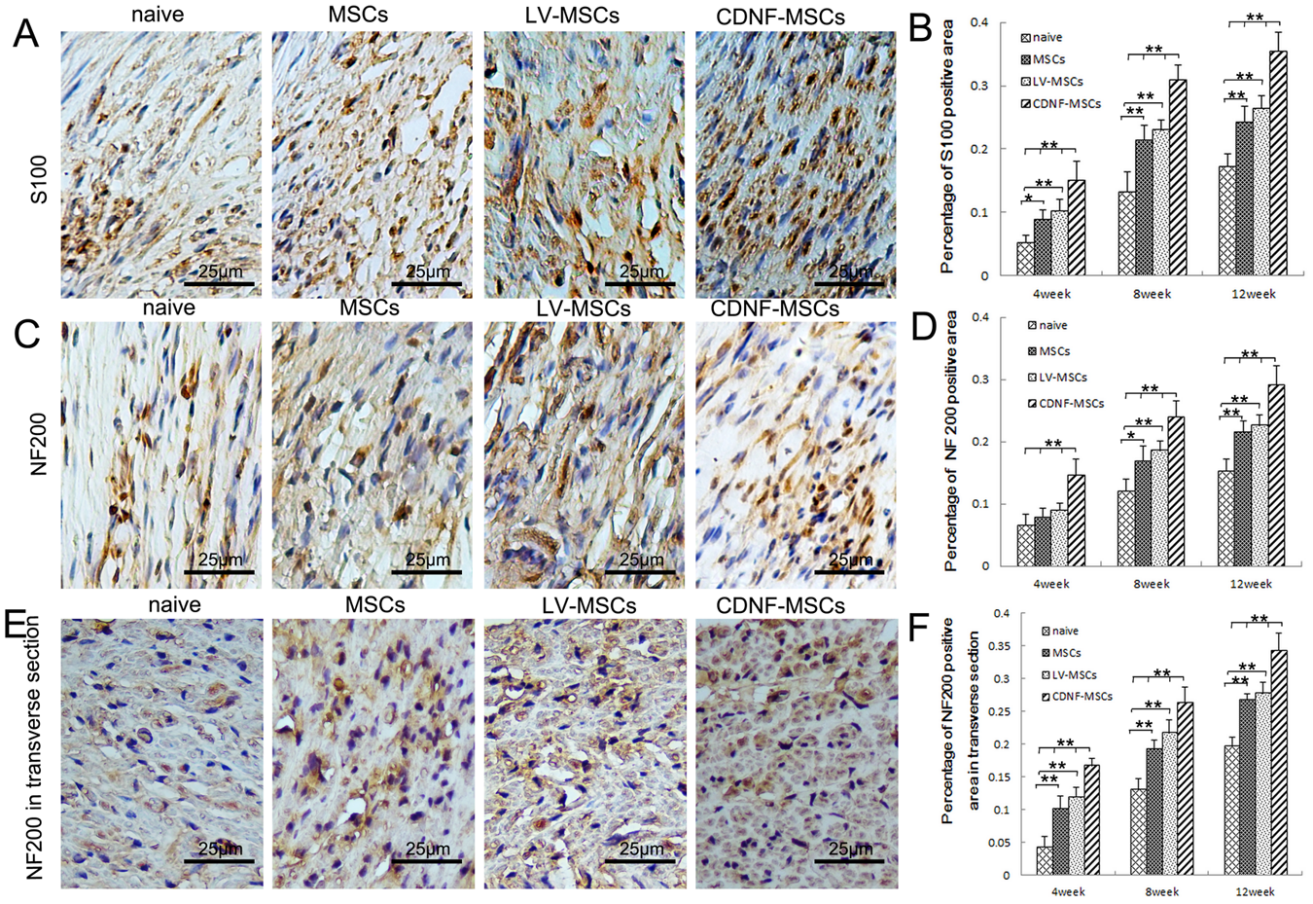
Histological analyses. (A and C) Longitudinal sections of the regenerated nerve stained with S100 and NF200 antibodies 12 weeks after repair. The S100-positive site is stained brown in A. The NF200-positive site is stained brown in B. Scale bar = 25 µm. (B and D) Quantitative analyses of the S100- and NF200-positive areas in each group. (E) Transverse sections of the regenerated nerve labeled with NF200 antibody 12 weeks after repair. The NF200-positive site is stained brown. Scale bar = 25 µm. (F) Quantitative analysis of the NF200-positive area in transverse sections from each group. n = 5, *p<0.05, **p<0.01.

Proximal and distal regenerating nerves were harvested at 4, 8, and 12 weeks after injury for histological analysis. S100 is a calcium-binding protein widely expressed in glial cells and is often used as a Schwann cell marker. Longitudinal sections were stained with S100, and the percentage of S100-positive area was statistically analyzed. Compared with the LV-MSCs, MSCs, and naive groups, the CDNF-MSCs group showed significantly greater S100 expression at 4, 8, and 12 weeks after injury (Fig. 4A, B). We also examined the area of NF200-positive axons in the longitudinal and transverse sections of the regenerated nerves. By this measure, the CDNF-MSCs group exhibited significantly better axonal regeneration compared with the other three groups at 4, 8, and 12 weeks after surgery (Fig. 4C–F).

### Remyelination of the regenerating sciatic nerve and walking track analysis

**Figure 5:**
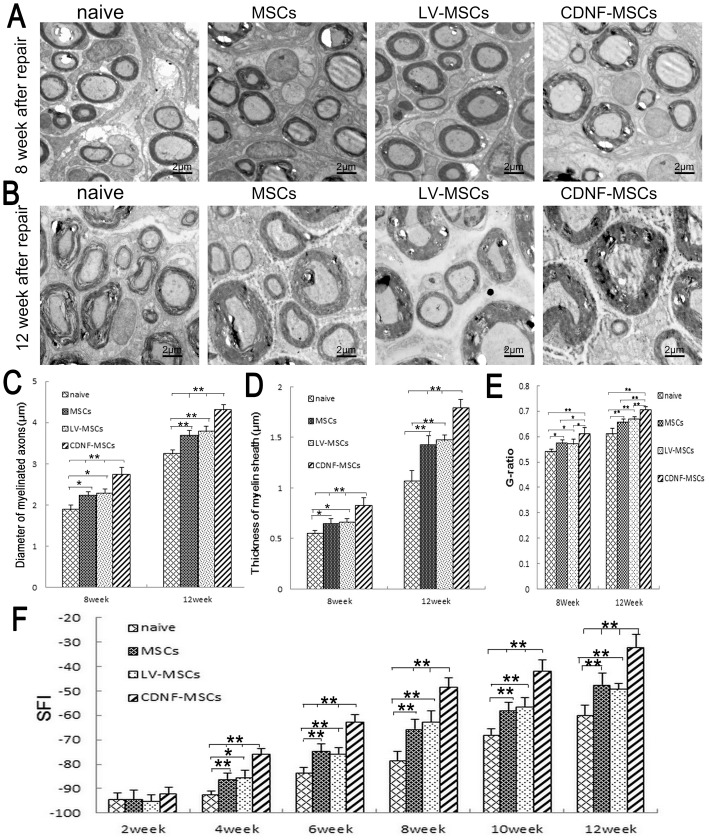
TEM of regenerating nerve and walking track analysis. (A and B) At 8 and 12 weeks after injury, ultrathin cross-sections of regenerating sciatic nerve were assessed with TEM. Scale bar = 2 µm. (C) Statistical analysis of myelinated axon diameters. (D) Statistical analysis of myelin sheath thicknesses. (E) Statistical analysis of the G-ratio. (F) Walking tracks were analyzed to assess motor function at 2, 4, 6, 8, 10, and 12 weeks after surgery. n = 5, *p<0.05, **p<0.01.

To detect myelin regeneration, the midpoints of the transplanted grafts were removed for TEM analysis, which further illustrated the myelinated nerve fiber morphology in the four groups. We used myelinated fiber thickness and transverse axonal diameter to evaluate sciatic nerve regeneration at 8 and 12 weeks after surgery. As shown in Fig. 5, the myelin sheaths appeared in cross-section in all of the transplanted groups. At weeks 8 and 12 after transection, the statistical analysis showed that the myelin sheaths thickness in the CDNF-MSCs group was significantly larger than in the MSCs, LV-MSCs and naive groups (Fig. 5D). The CDNF-MSCs group also had significantly greater axon diameters at 8 and 12 weeks after injury (Fig. 5C). In addition, G-ratio of the CDNF-MSCs group was significantly better than those of the other groups at 8 and 12 weeks after injury (Fig. 5E).

Rats were tested with walking track analysis to assess motor function recovery at 2, 4, 6, 8, 10, and 12 weeks after surgery. The sciatic function index (SFI) was used to compare the performances of the CDNF-treated and control groups. Walking track analysis showed spontaneous recovery of neurological function in the naive group as proved by the decreased SFI value. At weeks 4, 6, 8, 10, and 12 after surgery, the SFI scores for the CDNF-MSCs, MSCs, and LV-MSCs groups were significantly increased compared to the naive group, indicating that treatment with CDNF and MSCs accelerated locomotive function recovery of the severed sciatic nerve. Animals from the CDNF-MSCs group showed progressive improvements in motor function, as evidenced by SFI values, whereas there were no significant differences between the MSCs and LV-MSCs groups at 2, 4, 6, 8, 10, and 12 weeks after surgery (Fig. 5F).

### Survival of motor neurons

**Figure 6:**
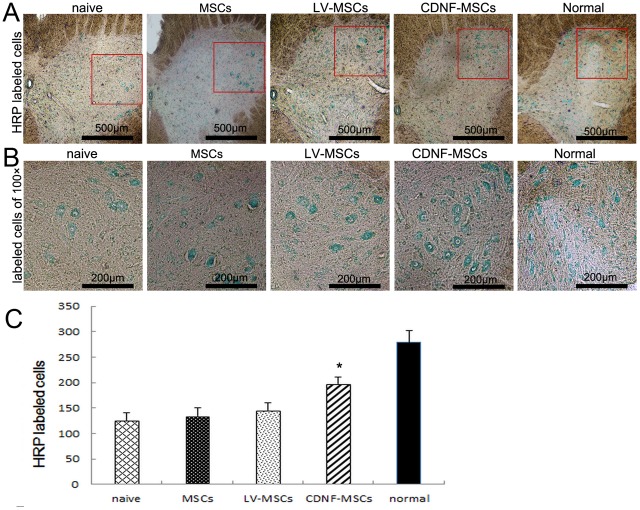
HRP tracing method showed motor neuron survival. (A) At 12 weeks after injury, 40-µm transverse sections of the lumbar spinal cord showing HRP-labeled neuron cell bodies. Scale bar = 500 µm. (B) HRP-labeled neuron cell bodies at 100×magnification. Scale bar = 200 µm. (C) Quantitative analysis of HRP-labeled neurons. n = 4, *p<0.05, **p<0.01.

The L4–5 segments were assessed to examine the effect of CDNF on neuronal survival. At 12 weeks after the surgery, an increased loss of spinal cord neurons in was observed in the naive group compared with the CDNF-treated group (Fig. 6A, B). The analysis shows that CDNF treatment significantly promoted the survival of motor neurons after nerve injury compared with the other three groups (Fig. 6C).

### Evaluation of amyotrophy

**Figure 7:**
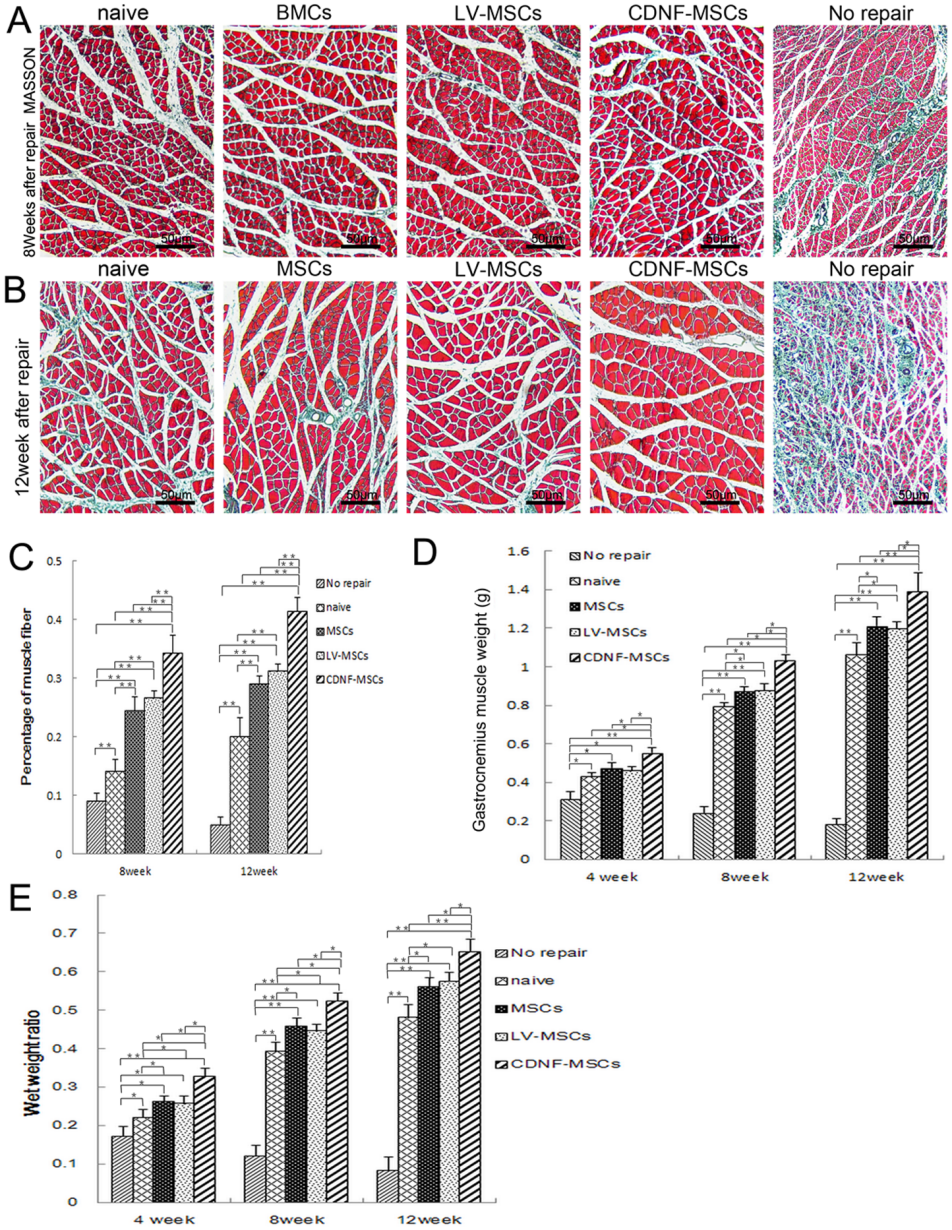
Evaluation of amyotrophy. (A and B) Masson’s collagen staining of sections of the transverse gastrocnemius muscle at 8 and 12 weeks after surgery. Scale bar = 50 µm (C) Quantitative analysis of the percentages of muscle fibers in each group. (D) Wet weight analysis of the gastrocnemius muscle at 4, 8 and 12 weeks after surgery. (E) Statistical analysis of the wet weight ratio of the gastrocnemius muscle. n = 5, *p<0.05, **p<0.01.

Masson’s collagen staining method directly showed the morphological changes in the gastrocnemius muscle at 8 and 12 weeks. The percentage of muscle fibers in the CDNF-MSCs group was significantly larger than those in the No repair, MSCs, LV-MSCs and naive groups at 8 and 12 weeks (Fig. 7C). The percentage of muscle fibers between the MSCs and LV-MSCs groups did not show obvious differences at 8 and 12 weeks after surgery (n = 5, P>0.05) (Fig. 7C). We also weighed the gastrocnemius muscles at 4, 8, and 12 weeks to assess muscle innervation recovery. At 8 and 12 weeks after surgery, the wet weights of the gastrocnemius in the CDNF-MSCs group were significantly higher than those in the No repair, LV-MSCs, MSCs, and DMEM groups, suggesting that treatment with CDNF-MSCs led to greater innervation of the gastrocnemius muscle (Fig. 7D). The wet weight ratio of gastrocnemius muscle in the CDNF-MSCs group was higher than superior to those in the No repair, LV-MSCs, MSCs, and DMEM groups (<0.05), but the difference between the LV-MSCs and MSCs groups was not statistically significant (>0.05). The wet weight of gastrocnemius muscle, the percentage of muscle fibers and the wet weight ratio of gastrocnemius muscle of all repair groups were better than those in the No repair group (<0.05).

## Discussion

Compared to the CNS, the microenvironment surrounding a PNS injury site provides greater potential for axonal regeneration. Optimizing this environment to promote better functional outcomes during nerve regeneration has been extensively studied. Autologous nerve transplantation has many shortcomings, which makes it necessary to identify new approaches.

With the rapid advances in tissue engineering technology, nerve conduits provide a potential alternative to sacrificing sound nerves for autologous grafts. Here, we employed a collagen nerve conduit to connect the distal and proximal nerves. After serving as a suitable scaffold to support axonal regeneration, the conduit eventually degraded, as confirmed by the SEM results before and after implantation. Degradation of the tube was confirmed through measuring the thickness of tube wall, which was reduced over time. Many flake defects on the surface developed as the tube degraded, implying ingrowth of nerve. Collagen exhibits excellent biocompatibility, which is effective at reducing inflammation of the surrounding wall. The collagen tube not only has enough strength and toughness to resist the pressure and collapse, but also has good flexibility and plasticity to provide a good basis for guiding and supporting nerve regeneration. These features also establish collagen as a superior material base for multidisciplinary and comprehensive treatment of nerve defect.

For many years, studies typically focused on the application of a single factor or method to improve the recovery of neurological function. However, satisfying functional recovery after PNI relies on many factors, including neuronal survival, the ability of the surrounding tissue to provide an effective support for regenerating axons, appropriate reinnervation, and the ability of the target organ to receive nerve reinnervation and recover from atrophy. Thus, the dynamic events occurring during regeneration require comprehensive strategies. In the present study, we designed a dual approach that combined CDNF-transduced MSCs with a collagen tube to provide trophic support and guide axon regeneration respectively.

Cell transplantation and MSC-delivered gene therapy to relieve nerve injury has become an important research direction in regenerative medicine. MSCs are multipotent cells with high plasticity, and they express many cytokines and ECM proteins, giving them the ability to support the repair of multiple tissues, including muscle, cartilage and fat [22], [23]. MSCs may promote nerve regeneration through many mechanisms, including the replacement of cells, synthesis of a variety of nutritional factors, the secretion of ECM molecules, the remodeling of the myelin sheath, maintenance of homeostasis, regulation of immune function after cell implantation. For PNI treatment, Schwann cells are considered to exert the best treatment effect; these myelinating cells of the PNS are critical for axon regeneration and re-myelination after injury. Compared with Schwann cells, MSCs are more readily accessible without the risk of damage to the nervous system, and they are less hampered by ethical issues because they can be autologously harvested.

The neuroprotective effect of CDNF has previously been demonstrated in rat experimental models of Parkinson’s disease [10], [24]–[26]. CDNF appears to promote nerve regeneration via neurotrophic and neuroprotective mechanisms. Like its homolog, the mesencephalic-astrocyte-derived neurotrophic factor (MANF), CDNF is a secreted protein with eight conserved cysteine residues, indicating a unique protein fold and defining a new, evolutionarily conserved protein family [25]. Mammals express CDNF and MANF, whereas invertebrates, such as *Caenorhabditis elegans* and *Drosophila melanogaster*, also possess a homologous gene for MANF/CDNF [26]. CDNF is widely expressed in several brain regions of adult mice, including the cortex, hippocampus, substantia nigra, cerebellum, and locus coeruleus [10]; however, CDNF levels are low in normal rat sciatic nerves [14]. Although the underlying mechanism remains to be elucidated and a CDNF receptor has not yet been identified, the structure of this protein suggests that its neurotrophic activity may reside in the N-terminal domain, whereas the C-terminal domain is involved in an endoplasmic reticulum (ER) stress response [24]. Consistent with this, early studies demonstrated that MANF rescues cerebral cortex neurons in a rat cerebral ischemia model, and CDNF alleviates damage to astrocytes caused by ER stress [11], [13]. CDNF protects microglia against inflammatory injuries and inhibits the production of proinflammatory cytokines by inhibiting JNK signaling [12]. Although much work has been done to elucidate the functions of CDNF, there is still much to be discovered concerning the mechanisms and actions of this neurotrophic factor.

In this study, we tested the hypothesis that MSC-based CDNF gene therapy enhances axonal regeneration and protects damaged neurons in a rat sciatic nerve injury model. Our results demonstrated that this method supported nerve regeneration across a 5-mm gap inside a collagen tube. After local injection of CDNF-MSCs, we detected high expression levels of recombinant CDNF. Immunohistochemical analyses for S100 and NF200 revealed better tissue organization in the CDNF-MSCs group, and ultrastructural analyses demonstrated significant differences among different treatment groups. The restoration of neural function and the consequent reduction of physical disabilities and complications is the primary goal of nerve injury research. However, direct evaluation of functional neural recovery in the rat is very difficult. Here, we tested functional recovery using indirect methods such as the change in gastrocnemius muscle weight and SFI values. Treatment with CDNF-MSCs dramatically reduced the muscle atrophy in the operated limbs, suggesting that is improved muscle activity and function. In addition, the CDNF-MSCs treated animals displayed a greater recovery of motor function at 2 weeks after grafting, implying that neural regeneration occurred earlier. Such regeneration, may be facilitated by the advantageous regenerative microenvironment, including the absorbability and supporting role of the collagen tube, significant neurotrophic and neuroprotective effects of CDNF, and the proliferation and secretion of cytokines and NTFs by MSCs.

In summary, we provide evidence that intranerve administration of LV-CDNF is useful for MSCs transduction, which effectively stimulated gene expression to promote axonal regeneration and motor neuron survival in a rat sciatic nerve defect model. Our findings raise the possibility of developing CDNF-transduced MSCs as a promising method for clinical peripheral nerve repair.
